# EHMTI-0297. Influences of psychiatric and behaviral factors on treatment effects in the school-age children with migraine

**DOI:** 10.1186/1129-2377-15-S1-G20

**Published:** 2014-09-18

**Authors:** K Lee

**Affiliations:** 1 Pediatrics, Hallym University, Seoul, Korea

## Purpose

School-aged children with severe migraine might need medication, and often accompanied by a psychiatric or behavioral problems. Thus, study for the effect of its problems on treatment of severe pediatric migraine is very important.

## Materials and methods

Among the 197 school-aged children who were diagnosed as migraine by ICHD-3b, seventy-two patients who need prophylactic therapy were enrolled from January 2012 to December 2013. Secondary headaches and headaches with ADHD or require psychiatric treatments were excluded. Before treatment, all patients were checked by Childhood behavioral checklist (CBCL), Children's Anxiety Scale (CAS) and Children's Depression Inventory (CDI) for screening of behavioral and psychological problems, and administered with topiramate (25-50 mg hs) as a prophylactic medication. They were followed-up at intervals of 2-4 weeks and grouped as good responder (GR) and poor responder (PR).

## Results

A total of 72 patients was M:F; 28:44, mean-age; 11.2±2.7 years. GR (45 patients; 62.5%, M:F;18:27, age; 10.7±2.5) and PR (27; 34.7%, M:F; 10:17, age; 12.0±3.0) were no statistically different. Features of headache in GR (duration; about 6 mo, attack duration; 9 h, frequencies; 12/mo) and PR (duration; about 12 mo, attack duration; 4 h, frequencies; 16/mo) had no significant difference. T-scores of GR and PR group were statistically different in total behavior problems, internalizing problems, depression/anxiety, social immaturity, attention problems of CBCL (p<0.01), but not different in factors of depression or anxiety in CAS and CDI.

## Conclusion

Because behavioral factors affect the treatment children with migraine, it is important to selection of drugs or management of behavioral problems.

No conflict of interest.

